# Supplementary material for: Deletion of wfs1 Impairs Oligodendrocyte Precursor Cells Dorsal Distribution and Myelination Through the wfs1-hmgcs1 Axis in Zebrafish
Source: Biology (Basel). 2026 Aug 16;15(16):1402. doi: 10.3390/biology15161402 (PMC13510151; doi:10.3390/biology15161402)
Supplement: Supplementary file 1 [file biology-15-01402-s001.zip › File S1-Transcriptome sequencing data and differentially expressed genes (DEGs) in wfs1-- zebrafish at 5 dpf/2.KEGG/wfs1KOvsWT/DOWN/src/dre04140.html]

dre04140


- dre:245702

- Down regulated genes

245702\_1(-1.45433245048201)

- dre:100000252

- Down regulated genes

100000252\_1(-1.7718817168434)

- dre:792689

- Down regulated genes

792689\_1(-1.78857482493481)

- dre:573492

- Down regulated genes

573492(-2.61487825195395)

- dre:792229

- Down regulated genes

792229(-6.97839628308154)

- dre:563140

- Down regulated genes

563140\_1(-1.16279481499233)

- dre:58100

- Down regulated genes

58100\_1(-4.1286149197908)

- dre:110438871

- Down regulated genes

321622(-1.53203125771205)

- dre:559564

- Down regulated genes

559564(-1.18025564761092)

- dre:100000242

- Down regulated genes

100000242(-6.34512572021214)

- dre:100000242

- Down regulated genes

100000242(-6.34512572021214)

Close
